# Evaluation of the reliability and validity of the health regulatory focus scale in Chinese samples

**DOI:** 10.3389/fpsyg.2023.1215209

**Published:** 2023-10-24

**Authors:** Xiaokang Lyu, Tingting Yang, Yanqin Fan, Haijuan Hong, Chunye Fu

**Affiliations:** ^1^Zhou Enlai School of Government, Nankai University, Tianjin, China; ^2^Songjiang District Central Hospital, Shanghai, China

**Keywords:** health regulatory focus scale, reliability and validity, psychometric properties, promotion focus, prevention focus

## Abstract

This study sought to validate the psychometric properties of the Health Regulatory Focus Scale (HRFS), emphasizing its manifestation and association with personality traits in a Chinese context. Originally developed by Ferrer, the HRFS gauges individuals’ inclinations either to avoid negative health outcomes (prevention focus) or achieve positive health outcomes (promotion focus). Our cross-sectional analysis involved a diverse sample of 652 Chinese participants, averaging 39.6 years in age (*SD* = 9.39). Data were analyzed using SPSS and AMOS, and both exploratory factor analysis (EFA) and confirmatory factor analysis (CFA) were employed to assess the HRFS’s factor structure. Additionally, we evaluated convergent and discriminant validity, criterion-related validity, internal consistency reliability, and test–retest reliability. The CFA results (CFI = 0.985, TLI = 0.971, RMSEA = 0.059, and SRMR = 0.047), combined with McDonald’s omega value (0.916) and the test–retest correlation coefficient (0.78) for the HRFS, underscore its robust construct validity and reliability. Furthermore, the promotion dimension of the HRFS exhibited significant positive correlations with all dimensions of the Chinese Adjectives Short Scale of Big-Five Factor Personality (BFFP-CAS-S). In conclusion, the HRFS’s Chinese adaptation offers a reliable and valid instrument for assessing health regulatory focus.

## Introduction

First proposed by Higgins (1997), regulatory focus theory distinguishes between two motivational orientations: promotion focus, associated with aspirations, advancement and ideals; and prevention focus, associated with responsibility, safety and security. One’s regulatory focus shapes behaviors and outcomes in various domains. For instance, research shows promotion focus associates with increased work engagement and innovative work behaviors (Jason and Geetha, 2021), while prevention focus links to reduced burnout (Lanaj et al., 2012). Regarding job crafting, promotion-focused employees craft more developmental opportunities (Sameer and Priyadarshi, 2021). In terms of creativity, Wang et al. (2021) found adolescents’ promotion focus positively predicted creative thinking. Beyond growing attention in organizational psychology, regulatory focus theory has also received attention in health contexts. Investigating regulatory focus in health behaviors could elucidate motivation processes for actions like screening, treatment adherence and lifestyle choices. Understanding these motivational mechanisms can ultimately inform tailored interventions to improve public health outcomes.

Health-related behaviors play a pivotal role in both individual wellbeing and public health outcomes. Modern individuals tend to seek full control over their health through various self-management strategies, including choosing nutritious foods, using oral health products, engaging in physical activity, and undergoing routine check-ups to achieve optimal wellbeing (Alpay et al., 2019; Kim et al., 2021). However, there exists a diversity in personal decisions and behaviors concerning the maintenance or enhancement of health status. For instance, some individuals are willing to try new therapies for potential health benefits (Aslam et al., 2020), while others are hesitant to receive flu vaccinations to avoid potential side effects (Rasul and Ahmed, 2023). Assessing an individual’s motivational style in relation to health behaviors proves instrumental in forecasting lifestyle choices that resonate with their intrinsic values and preferences (Mooradian et al., 2008; Ntoumanis et al., 2021).

Examining how regulatory focus affects health-related individual behaviors offers valuable insights into the determinants of individuals’ health-related choices. Similarly, health regulatory focus (HRF) pertains to an individual’s inclination to employ either promotion or prevention strategies in order to attain health-related goals (Gomez et al., 2013). Individuals oriented toward promotion focus are inclined to embrace behaviors that enhance their likelihood of realizing positive outcomes. Conversely, those with a prevention focus are more apt to adopt behaviors that mitigate the risk of adverse outcomes (Schmalbach et al., 2017). The shaping of HRF is multifaceted, influenced by an array of factors such as motivation (Scheerman et al., 2021; Westergren et al., 2021), personality traits (Sameer and Priyadarshi, 2020), cognitive styles (Higgins, 1998), and cultural background (Kruglanski et al., 2000).

Health Regulatory Focus has a broad application scope for studying various aspects of health-related individual behaviors, including disease prevention, health promotion, health communication, and health decision-making. It can investigate factors that influence individuals to adopt preventive measures such as vaccination (Ross, 2022), safe sex (Mao et al., 2021), or regular check-ups (Rodrigues et al., 2023). Additionally, HRF can identify factors that motivate health-promoting behaviors like exercise (Avraham et al., 2020), healthy eating (Lin and Yeh, 2017), and medication adherence (O’Connor et al., 2019). Furthermore, HRF can tailor health messages to an individual’s promotion or prevention focus, improving the effectiveness of health communication campaigns (Ludolph and Schulz, 2015). Lastly, HRF can help understand factors that influence an individual’s decision to seek medical care or adhere to treatment recommendations (Avraham et al., 2016).

Expanding upon the theoretical foundation established by Higgins (1997), as cited in Gomez et al. (2013), the conceptual framework of health regulatory focus has undergone significant development, leading to the emergence of Health Regulatory Focus Scale (HRFS). Notably, Gomez et al. (2013) formulated the HRFS as an instrument tailored to assess regulatory focus within health-specific domains. This scale encompasses both health promotion focus and health prevention focus dimensions. Subsequently, Ferrer et al. (2017) further refined the HRFS, crafting a concise yet robust 12-item measure that comprises distinct promotion and prevention subscales, thereby capturing the overarching construct of general health regulation focus.

Ferrer et al. (2017) conducted a comprehensive validation study to ascertain the psychometric properties of the HRFS. Their investigation revealed favorable outcomes in terms of internal consistency, test–retest reliability, and construct validity. These outcomes solidify the credibility of the HRFS as a reliable instrument for assessing health regulatory focus. While the HRFS has garnered international application, extending beyond national borders to countries such as the United States, the United Kingdom, Italy, and China (Catellani et al., 2021; Ferrer et al., 2021; Kim and Kim, 2022; Zhao et al., 2022; Nurek and Kostopoulou, 2023), it is noteworthy that the scale’s psychometric evaluations have hitherto been confined to the U.S. context.

Given the growing emphasis on culturally nuanced research, it is imperative to extend the evaluation of the HRFS to diverse cultural settings. This endeavor is essential to establish the cross-cultural applicability of the HRFS and facilitate any necessary adaptations. Cultural disparities in lifestyles and health behaviors (Lee and Shin, 2013) underscore the need to discern whether the patterns of health regulatory focus observed in the Chinese populace align with those identified in other cultural contexts. Hofstede’s cultural dimensions theory proposes that national culture influences values and behaviors (Hofstede et al., 2010), including individualism vs. collectivism, traditionalism, long-term orientation, and indulgence vs. restraint (Bonello et al., 2018). China exhibits a highly collectivist culture marked by strong social structures and family loyalty, contrasting individualistic Western societies. Moreover, China ranks low in indulgence, reflecting a restrained culture focused on moderation rather than freely pursuing enjoyment. These dimensions indicate key cultural differences that may shape health motivations and necessitate validating the HRFS in China specifically. For instance, China’s collectivism may link to greater prevention focus, emphasizing in-group duties vs. promotion-focused Western cultures. However, China’s modernizing economy could also shift values toward promotion over tradition. Examining regulatory focus in China may reveal nuances between indigenous and emerging values. Adapting the HRFS to align with Chinese culture is essential for understanding how to effectively promote public health in this context. Consequently, a rigorous examination of the psychometric properties of the HRFS, specifically within the Chinese cultural milieu, is both warranted and timely.

Moreover, exploring the intricate relationship between health regulatory focus and personality traits is a promising area of inquiry. Previous research has suggested links between health regulatory focus and specific personality traits, such as the association between higher conscientiousness and promotion focus, and higher neuroticism and prevention focus (Higgins, 1997; Sameer and Priyadarshi, 2020). However, understanding the interplay between health regulatory focus and personality traits necessitates cross-cultural investigation to uncover potential cultural influences. It is important to note that the relationship between health regulatory focus and personality traits within the Chinese context remains unclear and requires further examination. By elucidating the alignment between health regulatory focus and personality traits within the Chinese cultural framework, we can gain valuable insights to develop effective health promotion strategies tailored to this context. A comprehensive exploration of the cultural dynamics influencing health motivation has the potential to inform targeted health policies and campaigns.

The primary objective of this study is to comprehensively examine and validate the cross-cultural applicability of the Health Regulatory Focus Scale (HRFS) within the context of diverse populations, with a particular focus on its manifestation and interrelation with personality traits in China. By addressing these objectives, the study endeavors to contribute essential insights into the utility of the HRFS for culturally tailored health interventions and policies, enhancing our understanding of the interplay between health regulatory focus and personality across diverse cultures.

## Materials and methods

### Participants and procedure

This project was a cross-sectional study using a non-probabilistic sample of the general population. In this study, we recruited 764 participants through social networks and collected data using wenjuan.com, a free questionnaire survey platform. Online data collection enables broader reach, accessibility to diverse participants, and cost and time efficiency compared to traditional offline methods. Participants provided online informed consent and the study was approved by the Biomedical Ethics Committee of Nankai University. After excluding invalid questionnaires, 652 adults with a mean age of 39.6 years (*SD* = 9.39; range = 18∼78 years) participated, with 66.1% being female (*n* = 431). Participants had varying education levels, with 10.3% (*n* = 67) reporting junior middle school or below, 10.3% (*n* = 67) reporting senior middle school or technical secondary, 60.4% (*n* = 394) reporting junior college or undergraduate, and 19.0% (*n* = 124) reporting a master’s degree. Table 1 presents information on working status, marital status, self-assessment of health status, and fertility status. After a 3-month interval, 89 valid questionnaires were collected from the 100 participants who underwent retesting.

**TABLE 1:** Descriptive statistics for the participants (*N* = 652).

Variables	Total sample *N* (%)
**Age (years)**	
≤29	79 (12.1)
29–39	295 (45.2)
39–49	193 (29.6)
49–59	57 (8.74)
≥59	28 (4.36)
**Sex**	
Male	221 (33.9)
Female	431 (66.1)
**Educational level**	
Junior middle school or below	67 (10.3)
Senior middle school or technical secondary	67 (10.3)
Junior college or undergraduate	394 (60.4)
Master’s degree	124 (19.0)
**Working status**	
Medical worker	260 (39.9)
No medical worker	390 (60.1)
**Marital status**	
Unmarried	76 (11.7)
Married	552 (84.7)
Others	24 (3.6)
**Self-assessment of health status**	
Very poor	22 (3.37)
Poor	59 (9.06)
Neutral	297 (45.6)
Quite good	252 (38.6)
Very good	22 (3.37)
**Fertility status**	
No children	118 (18.1)
A child	401 (61.5)
Two children	125 (19.2)
Three or more children	8 (1.2)

### Measures

#### Health regulatory focus scale (HRFS)

Ferrer et al. (2017) developed the Health Regulatory Focus Scale (HRFS) to evaluate individuals’ tendencies to avoid negative health outcomes (prevention focus) or achieve positive health outcomes (promotion focus). The HRFS comprises twelve items, with six promotion focus and six prevention focus items in each subscale. Respondents rate their answers on a seven-point scale, ranging from 1 (“strongly disagree”) to 7 (“strongly agree”). The scale score is calculated by averaging the items, with items 1–6 reflecting promotion focus and items 7–12 reflecting prevention focus. A higher score indicates a greater likelihood of adopting the corresponding regulatory focus strategy. In previous studies, Cronbach’s α values for the prevention focus and promotion focus ranged from 0.70 to 0.85 and from 0.83 to 0.89, respectively (Ferrer et al., 2017), indicating good internal consistency. In this study, the Cronbach’s α values for promotion focus and prevention focus were 0.84 and 0.92, respectively, suggesting high reliability.

#### Chinese adjectives short scale of big-five factor personality (BFFP-CAS-S)

The Chinese Adjectives Short Scale of Big-Five Factor Personality (BFFP-CAS-S), developed by Luo and Dai (2018), assesses the Big-Five personality traits. The scale includes 20 items selected from BFFP-CAS, with each of the five dimensions having four items. The dimensions measured by BFFP-CAS-S are Extraversion (items 1, 6, 11, and 16), Agreeableness (items 2, 7, 12, and 17), Conscientiousness (items 3, 8, 13, and 18), Neuroticism (items 4, 9, 14, and 19), and Openness (items 5, 10, 15, and 20), where items 4, 9, 14, and 19 are reverse-scored. Utilizing a 6-point scale, ranging from 20 to 120 total points. A higher score signifies a stronger correspondence of personality traits with the five specified dimensions. The internal consistency of each dimension ranged from 0.72 to 0.85.

#### Translation procedure of the HRFS

The Health Regulatory Focus Scale (HRFS) was translated into Chinese utilizing a rigorous double translation and back-translation method. Initially, two graduate students in psychology independently translated the English version into Chinese. Subsequently, a Chinese journal editor and an evidence-based psychology professor collaborated to integrate these translations into a cohesive draft. Two English teachers, both with experience teaching in English-speaking countries, then jointly translated the Chinese version back into English and compared it with the original text. They confirmed that the translated English version was congruent with the original, preserving its essential meaning and content. The finalized Chinese version of the HRFS was subsequently employed to assess health regulatory focus within a Chinese sample.

#### Data analysis

The study employed SPSS version 25 for descriptive statistical analysis, including Student’s *t*-tests, Pearson’s *r* correlations, *F*-tests, and internal consistency tests. AMOS 24 was utilized for exploratory and confirmatory factor analyses. Descriptive statistics were applied to examine participant characteristics. The study also used an *F*-test to analyze demographic differences in HRFS. The Tukey’s HSD method was employed for *post-hoc* testing to ascertain group distinctions in education levels across the two dimensions of health regulatory focus. This approach aimed to uncover the potential rationales behind the disparate health regulatory focus between individuals with and without a university degree.

The sample was randomly split into two halves for exploratory factor analysis (EFA) and confirmatory factor analysis (CFA). EFA was conducted on the first half of the sample (*n* = 326), and CFA was performed on the second half of the sample (*n* = 326). The data’s suitability for EFA was assessed using the Kaiser-Meyer-Olkin (KMO) test and Bartlett’s test of sphericity. Construct validity was evaluated with principal component analysis and Varimax rotation (Brown, 2015; Watkins, 2018). The goodness of fit for CFA was analyzed using Chi-square (χ^2^), standardized Chi-square/df (χ^2^*/df*), Comparative Fit Index (CFI), Tucker-Lewis Index (TLI), Root Mean Squared Error of Approximation (RMSEA), and Standardized Root Mean Square Residual (SRMR). RMSEA and SRMR values of ≤0.08 indicate acceptable model fit, while values of ≤0.05 indicate good model fit, and CFI and TLI values of ≥0.90 indicate adequate model fit, as per conventional guidelines (Hu and Bentler, 1998; Marsh et al., 2004; Brown, 2006).

Convergent validity was assessed using Construct Reliability (CR) and Average Variance Extracted (AVE). Discriminant validity was assessed using the square root of AVE and Pearson’s correlation coefficient. Criterion-related validity was evaluated using Pearson’s correlation coefficient. If this value is of the square root of AVE higher than the correlation between the factor and other factors of the model, the factor is considered independent of others (Teo et al., 2009).

The reliability of the sample was evaluated using McDonald’s omega for internal consistency. A McDonald’s omega value of 0.6 to 0.7 is minimally acceptable, 0.7 to 0.8 is respectable, and more than 0.8 is excellent (Zinbarg et al., 2005). Another approach used to assess reliability was to determine the stability over time (Kramer and Feinstein, 1981). This was achieved by calculating the correlation coefficient between the baseline test and the retest conducted 3 months later. A correlation >0.81 was considered “excellent,” 0.61 to 0.80 “good,” 0.41 to 0.60 “moderate” and <0.40 no correlation (Landis and Koch, 1977).

## Results

### Construct validity test

Based on a KMO value of 0.905 and a significant χ^2^ value of 5,104.33 (*p* < 0.001) in Bartlett’s test of sphericity, the data were deemed appropriate for factor analysis. An eigenvalue greater than or equal to 1.0 was selected as the cutoff for conducting EFA. Factor loadings ranging from 0.59 to 0.89 were observed, meeting the criterion of being above 0.30 but not approaching 1.0. EFA results (Table 2) revealed two factors, explaining 66.8% of the variance and falling within the recommended range of 50.0–60.0% for scale descriptive power (Hair et al., 2009).

**TABLE 2:** Exploratory factor analysis of HRFS and convergent validity.

No	Item	FL	Estimate	AVE	CR
**Factor 1: promotion focus**					
1	I frequently imagine how I can achieve a state of “ideal health.”	0.59	0.57	0.51	0.86
2	I think of good health as a key to a happy life.	0.69	0.61		
3	Doing healthy things gives me a sense of accomplishment.	0.86	0.86		
4	When I engage in healthy behaviors, I am pleased with myself.	0.86	0.86		
5	I would do anything to maintain a good, healthy body.	0.71	0.69		
6	I admire people who do things that make them very healthy.	0.68	0.62		
**Factor 2: prevention focus**					
7	I often worry that I am not doing the best I can to improve my health.	0.61	0.68	0.68	0.93
8	I often imagine myself being ill in the future.	0.80	0.73		
9	I am anxious that I am not following through on my obligations and being as responsible as I should about taking care of my health.	0.86	0.85		
10	When I see people who are very sick because they did not take care of their health, I get scared thinking that could be me in the future.	0.88	0.88		
11	I often worry about not feeling as healthy as I used to be.	0.86	0.87		
12	Thinking about my health usually makes me worry.	0.89	0.89		

FL, factor loading; AVE, average variance extracted; CR, construct reliability.

This study analyzes the validation factors of the second half of the sample (*n* = 326) and labels each factor as “promotion focus (Factor 1)” and “prevention focus (Factor 2).” The model fit indicators reported by AMOS software are robust, being less susceptible to sample size, model misspecification, and model complexity. The goodness-of-fit of the model was assessed using CFA, with 12 items assigned to two factors. The CFI and TLI indices were 0.985 and 0.971, respectively, indicating a good model fit. The RMSEA and SRMR values were 0.059 and 0.047, respectively, which meet the acceptable level of model fitting (Table 3).

**TABLE 3:** Confirmatory factor analysis of HRFS (*N* = 326).

	χ^2^	*df*	χ^2^/*df*	CFI	TLI	RMSEA	SRMR
HRFS	114	35	3.257	0.985	0.971	0.059	0.047

CFI, comparative fit index; TLI, Tucker–Lewis index; RMSEA, root mean squared error of approximation; SRMR, standardized root mean square residual.

The CR values for testing the convergent validity of the final model ranged from 0.86 to 0.93, with AVE values of 0.51 to 0.68, meeting the recommended thresholds of a CR of above 0.70 and AVE of above 0.50 (Table 2). Discriminant validity was tested by comparing the square root of AVE with the correlation coefficients of each factor (Table 4). The square root of AVE ranged from 0.71 to 0.82, and correlation coefficients ranged from 0.50 to 0.68, indicating that discriminant validity was established.

**TABLE 4:** Descriptive statistics and correlations between the HRFS and BFFP-CAS-S.

Variables	*M* ± *SD*	*r*	
		**HRFS-promotion focus**	**HRFS-prevention focus**
HRFS-promotion focus	34.38 ± 6.02	0.71^1^	-
HRFS-prevention focus	28.09 ± 9.53	0.50***	0.82^1^
BFFPCASS-extraversion	16.15 ± 4.76	0.31***	0.07
BFFPCASS-agreeableness	18.31 ± 4.27	0.27***	0.001
BFFPCASS-conscientiousness	17.14 ± 4.18	0.37***	0.09**
BFFPCASS-neuroticism	11.36 ± 4.33	−0.35***	−0.03
BFFPCASS-openness	15.28 ± 4.57	0.26***	0.07

**p < 0.01. ***p < 0.001. ^1^Square root AVE.

### Criterion-related validity

Overall, the score of promotion focus dimension of HRFS and the scores of all dimensions of BFFP-CAS-S were significantly correlated. Among them, the promotion focus factor was strongly positive correlated with traits of extraversion, agreeableness, conscientiousness, and openness, and a significant negative correlation with neuroticism. The prevention focus factor had a low and significant correlation with the conscientiousness, but had low and non-significant correlations with the other traits of Big-Five Factor Personality (Table 4).

### Internal consistency reliability

The McDonald’s omega for the entire HRFS was 0.916, while the promotion focus and prevention focus dimensions had McDonald’s omega of 0.890 and 0.940, respectively. All values were above 0.8, indicating good internal consistency and stability of the HRFS.

### Test–retest reliability

The test–retest reliability of the HRFS was assessed by administering the test to 89 participants after a 3-month interval. The results indicated optimal test–retest reliability, with correlation coefficients of 0.78 for the total scale and 0.77 and 0.76 for the two dimensions, indicating good retest reliability.

### Descriptive statistics and differences based on demographic variables

Table 4 displays descriptive statistics for the 2 and 5 dimensions of the HRFS and BFFP-CAS-S scales in the total sample. Differences in the promotion and prevention dimensions were significant across education levels (Table 5). *Post-hoc* tests (Table 6) showed that there was no significant difference in either the promotion or prevention focus at the junior middle school or below and senior middle school or technical secondary; the same results were obtained at the junior college or undergraduate and master’s degree. These results suggest that, in terms of regulatory focus, individuals within these educational groups exhibited similar tendencies. However, the junior college or undergraduate and master’s degree participants had significantly higher promotion and prevention focus scores than the junior middle school or below and senior middle school or technical secondary. Our analysis revealed that there was no statistically significant difference in total scores between medical workers and non-medical workers in relation to the promotion and prevention dimensions of health regulatory focus.

**TABLE 5:** Differences in educational level in promotion focus and prevention focus dimensions.

Variables	Group	*N*	*M*	*SD*	*F*	*p*	η^2^
Promotion focus	Junior middle school or below	67	36.50	6.54	8.36	<0.001	0.04
	Senior middle school or technical secondary	67	36.50	5.70			
	Junior college or undergraduate	394	34.10	6.07			
	Master’s degree	124	33.00	5.18			
Prevention focus	Junior middle school or below	67	31.20	9.08	8.67	<0.001	0.04
	Senior middle school or technical secondary	67	31.40	9.50			
	Junior college or undergraduate	394	27.90	9.67			
	Master’s degree	124	25.40	8.38			

**TABLE 6:** *Post hoc* comparisons amonge different educational levels in promotion focus and prevention focus dimensions.

Promotion focus					Prevention focus		
**Group A**	**Group B**	***t***	***p***	**Group A**	**Group B**	***t***	***p***
1	2	0.044	1.000	1	2	−0.111	1.000
	3	3.128	0.010		3	2.678	0.038
	4	3.939	<0.001		4	4.080	<0.001
2	3	3.071	0.012	2	3	2.823	0.025
	4	3.889	<0.001		4	4.206	<0.001
3	4	1.785	0.281	3	4	2.570	0.051

1 = Junior middle school or below. 2 = Senior middle school or technical secondary. 3 = Junior college or undergraduate. 4 = Master’s degree; group A/B allows two-by-two comparisons.

## Discussion

The Health Regulatory Focus Scale (HRFS) serves as a vital instrument in crafting effective interventions for health behavior change. This study was designed to validate and ascertain the reliability of the HRFS within the context of Chinese culture. Participants were recruited nationwide in mainland China through social networks, and comprehensive statistical analyses were conducted. The findings reveal that the HRFS possesses robust construct validity, maintaining an identical bifactor structure to the original English version. Additionally, the score of the promotion focus dimension of the HRFS exhibited a significant correlation with the scores across all dimensions of the BFFP-CAS-S. The HRFS further demonstrated satisfactory convergent and discriminant validity, internal consistency, and retest reliability. Consequently, the HRFS emerges as a reliable and valid tool for assessing health regulatory focus in China, and it holds potential for effective utilization in the development of behavior change interventions.

Following the execution of exploratory factor analysis (EFA), confirmatory factor analysis (CFA), convergent validity testing, and discriminant validity testing, the Health Regulatory Focus Scale (HRFS) was finalized with two distinct factors: “promotion focus” and “prevention focus,” thereby affirming its construct validity. The study substantiated that the two-factor model of the HRFS was congruent with the data, with EFA revealing pronounced associations between all items and their corresponding factors. Furthermore, the CFA indicated that the model fitting index met acceptable standards. In assessing convergent validity, the results confirmed that the model could reliably gauge the intended constructs. Discriminant validity analysis further demonstrated that the measures within the model were discrete and not excessively correlated. Collectively, these findings validate the finalized model, attesting to its capability to accurately measure HRFS constructs. HRFS-Promotion and HRFS-Prevention are conceptualized as types of regulatory focus that can influence health behavior. In line with this understanding, we hypothesized that one or both of these factors would exhibit a connection to behavioral intentions (Lalot et al., 2019; Ku et al., 2022).

To further examine the usefulness of the HRFS, criteria validity was considered in the current study. Previous research has consistently shown a positive correlation between conscientiousness and both prevention and promotion focus (Wallace and Chen, 2005; Liu et al., 2020). Conscientious individuals tend to prioritize their health through proactive health strategies, indicating alignment with a promotion focus. Simultaneously, their cautiousness toward potential risks is associated with a concurrent development of a prevention focus, driving them to adopt safer behaviors. Furthermore, our research findings align with those of Higgins et al. (2001), which also reported a positive correlation between extraversion and promotion focus. Extraversion, characterized by sociability, assertiveness, and a preference for novel experiences, suggests that individuals with higher extraversion levels may be more inclined to pursue health goals related to achievement and personal advancement. However, it’s crucial to note that this positive correlation does not extend to prevention focus, which centers on avoiding negative health outcomes and maintaining health through precautionary measures. The absence of a significant correlation between extraversion and prevention focus in this context suggests that extraversion may not strongly influence individuals’ tendencies toward health risk aversion or safety-conscious health behaviors. Similarly, Ouschan et al. (2007) found a negative correlation between neuroticism and promotion focus, which is consistent with our findings. However, no significant correlation was observed between neuroticism and prevention focus, supporting our results. These findings hold practical implications for health interventions and promotion strategies. For instance, recognizing that individuals with higher extraversion levels may be more receptive to health messages emphasizing achievement and personal growth in health-related goals can guide tailored health communication strategies. It is important to acknowledge that regulatory focus can manifest as either a stable personality trait or a temporary motivational tendency influenced by self-regulatory experiences and situational factors (Geers et al., 2005). Future research may delve into the underlying mechanisms of this relationship and explore how it can be leveraged to enhance public health initiatives.

This study used McDonald’s omega to establish the internal consistency reliability of the Health Regulatory Focus Scale (HRFS). The HRFS showed high internal consistency (omega = 0.916) and moderate test–retest reliability (*r* = 0.78; factor-level *r* > 0.76). This study’s contribution is the analysis of previously lacking internal consistency and test–retest reliability of the HRFS (Schmalbach et al., 2020), confirming its reliability in measuring health regulatory focus.

The findings pertaining to disparities in health regulatory focus based on demographic variables, particularly education level, unveil an intriguing nexus between educational attainment and the orientation toward health regulation. Notably, a discernible trend emerged, wherein heightened levels of education exhibited an inverse relationship with both health promotion and prevention focus. However, this trend did not extend to a distinction between the two highest and two lowest strata of educational attainment. A plausible rationale underlying these observations could be ascribed to the enriching influence of higher education, endowing individuals with the requisite knowledge for making judicious health-related decisions. This heightened informational capacity could potentially attenuate the reliance on innate motivational proclivities, whether they manifest as promotion or prevention focus. This premise is supported by prior research (Sarhan et al., 2021), which posits that individuals with greater educational exposure possess elevated health literacy and cognitive reservoirs, consequently mitigating the need for habitual reliance on motivational orientation. In contrast, individuals with comparatively limited educational backgrounds may exhibit a heightened dependence on broader regulatory frameworks to steer health behaviors. This phenomenon underscores the interplay between educational attainment, cognitive resources, and motivational tendencies, thereby accentuating the need for further exploration into the nuanced mechanisms underpinning health regulatory focus within distinct educational strata. These findings hold practical significance for health professionals, policymakers, and educators. They underscore the importance of comprehending how individuals’ health regulatory focus changes across various educational levels, guiding the design of customized health education initiatives. Furthermore, exploring the impact of health regulatory focus on health-related behaviors and outcomes in diverse educational settings can yield valuable insights with practical applications.

An interesting finding of this study was the lack of a significant association between profession (medical vs. non-medical) and health regulatory focus. In our experience, physicians may be inclined toward higher prevention focus due to their emphasis on risk and adherence to protocols. However, this study did not find significant differences in regulatory focus between medical and non-medical professionals. One potential reason is that regulatory focus is shaped more by cultural or personality factors rather than occupation. There may also be diversity within the medical profession itself that obscures differences compared to other professions. Further research is needed to understand the drivers of regulatory focus in relation to profession. Investigating this question across cultural contexts and specific medical specialties could provide useful insights. Though no link was found here, future studies can continue to explore potential professional influences on health regulatory focus in Chinese and other populations.

In subsequent research, the validated Chinese version of the HRFS can serve as a tool to examine the relationship between regulatory focus and health outcomes within Chinese populations. This exploration may encompass an analysis of how regulatory focus influences health behaviors, attitudes, and outcomes across various Chinese subpopulations. Interventions specifically targeting individuals’ regulatory focus orientation could be assessed for their efficacy in enhancing health outcomes within the Chinese cultural context. Healthcare providers may also leverage an understanding of individuals’ regulatory focus orientation to tailor interventions, thereby more effectively addressing patients’ unique needs and preferences. A comparative analysis between findings from the Chinese sample and those from other cultural backgrounds could further contribute to assessing the cross-cultural generalizability of the HRFS. Overall, the application of the HRFS within a Chinese setting holds the potential to yield valuable insights into the regulatory focus of Chinese individuals concerning health. Such research can significantly enrich our comprehension of cross-cultural variations in health behavior, offering a nuanced perspective that may inform both clinical practice and public health policy.

This study validated an HRFS scale with a detailed construct of two factors that is better suited for regulatory focus in the health field. The HRFS retained all the items from the original American health regulatory focus version, and its structure composition was unchanged. The HRFS enables a better understanding of how health promotion and health prevention focus impact an individual’s response to health programs or treatments, allowing researchers and practitioners to customize interventions to meet recipients’ specific needs.

## Limitations

The limitations of this study must be acknowledged in the interpretation of the findings. The sample obtained cannot be deemed representative of the Chinese population for two primary reasons. First, despite the sufficiency in the number of samples, the gender distribution is skewed, with more women than men, leading to an under-representation of the male demographic in our sample. Second, the educational level within the sample is predominantly high, with the majority of participants possessing at least a junior college education. This concentration of higher education levels may introduce a selection bias. Future research should endeavor to evaluate the performance of the HRFS within more diverse and representative samples to validate and potentially generalize these findings. Such efforts would contribute to a more robust understanding of the scale’s applicability across various demographic and educational strata.

Another important consideration is whether the HRFS validated in this Chinese sample demonstrates applicability in other cultural contexts. On one hand, the scale’s sound psychometric properties and factor structure in this collectivist, restrained culture suggests it may transcend some cultural variations. The underlying dimensions of promotion and prevention focus may represent universal motivational mechanisms. However, it is also possible that the relative strength of each orientation and specific manifestations could differ across individualist and indulgent societies. For instance, the scale items may need adjustment for cultures with higher promotion focus. Future research should examine the scale in diverse cultural settings to determine its boundaries.

## Conclusion

The Chinese version of HRFS contributed a reliable and valid measure of health regulatory focus. The two-factorial structure assumption showed a good model fit, and the HRFS exhibited satisfactory convergent and discriminant validity, internal consistency, and retest reliability. This scale is suitable to serve as a screening tool in health-related contexts. Therefore, we can reaffirm the original recommendation for the HRFS as a tool for psychological research on health behavior and motivation.

## Data availability statement

The original contributions presented in the study are included in the article/supplementary material, further inquiries can be directed to the corresponding author.

## Ethics statement

The studies involving humans were approved by the Biomedical Ethics Committee of Nankai University (NKUIRB2022103). The studies were conducted in accordance with the local legislation and institutional requirements. The participants provided their written informed consent to participate in this study.

## Author contributions

XL, TY, and CF contributed to data analysis, research design, and manuscript preparation. YF and HH validated the translation version of the HRFS and recruited participants. CF proofread the manuscript and assisted with fundraising. All authors made significant contributions to this article and have approved the final version for submission.
